# Adenoid cystic carcinoma of the retromolar pad region: A case report

**DOI:** 10.1016/j.ijscr.2024.109567

**Published:** 2024-03-20

**Authors:** Zixian Xu, Canbang Peng, Yuhao Zhang, Lizhong Chen, Jiemei Zhai

**Affiliations:** a Department of Basic Science of Stomatology, School of Stomatology Kunming Medical University, 1168 Chunrong West Road, Chenggong Zone, Kunming 650500, People's Republic of China; b Department of Oral and Maxillofacial Surgery, School of Stomatology Kunming Medical University, 1088 Middle Haiyuan Road, High-Tech Zone, Kunming 650106, People's Republic of China; c Department of Oral Medicine, Stomatological Center of the First People's Hospital of Yunnan Province, 157 Jinbi Road, Xishan Zone, Kunming 650034, People's Republic of China; d Department of Oral Medicine, School of Stomatology Kunming Medical University, 1088 Middle Haiyuan Road, High-Tech Zone, Kunming 650106, People's Republic of China; e Department of Basic Science of Stomatology, School of Stomatology Kunming Medical University, 1088 Middle Haiyuan Road, High-Tech Zone, Kunming 650106, People's Republic of China

**Keywords:** Adenoid cystic carcinoma, Retromolar pad, Differential diagnosis, Long-term follow-up, Case report

## Abstract

**Introduction:**

Adenoid cystic carcinoma (ACC) is one of the most common salivary gland malignancies, mostly occurs in the major and minor salivary glands in the oral and maxillofacial region. The development of ACC in the retromolar pad is extremely rare, which limits establishing proper diagnosis and management.

**Presentation of case:**

A patient described a 2-month history of finding a mass behind the lower left posterior teeth. Based on the physical examination and radiographic findings, we got an initial impression of a benign mucocele, the nature of which was to be investigated further. Pathological examination of the resected tissue resulted in a diagnosis of ACC. Follow-up visits showed no recurrence during the subsequent 54 months.

**Discussion:**

In cases with an uncertain diagnosis based on medical history, clinical features and imaging examinations, it is important to proceed carefully with the possibility of a tumor in mind.

**Conclusion:**

ACC in the retromolar pad is rare and can be easily misdiagnosed. Clinical, radiographic, and pathological evidence confirm a definitive diagnosis. Long-term follow-up is important for the full analysis of ACC treatment.

## Highlights

• Adenoid cystic carcinoma is one of the most common malignant tumors of the salivary gland.
• Adenoid cystic carcinoma in the retromolar pad has not been reported and easily misdiagnosed.
• Given the prognosis of ACC, actively treatment of the primary lesion and thorough follow-up are necessary.

## 1 Introduction

Adenoid cystic carcinoma (ACC) is one of the most common salivary gland malignancies, mostly occurs in the major and minor salivary glands in the oral and maxillofacial region, with more than half occurring in the parotid and submandibular glands, and the palate is the most commonly affected site [1]. ACC represents 1 % of head and neck cancers and 10 % of salivary gland tumors. ACC is characterized by slow growth, strong invasiveness and distant metastasis [2]. To date, ACC developing in the retromolar pad has not been reported in literature. This paper presents a rare case of ACC in a 59-year-old female originated from the minor salivary glands in the retromolar pad, intending to provide a reference to avoid the clinical misdiagnosis of ACC in uncommon sites and report its long-term prognosis after treatment. The work has been reported in line with the SCARE 2020 criteria [3].

## 2 Presentation of case

A 59-year-old female patient visited the Affiliated Stomatology Hospital of Kunming Medical University in June 2019 with a chief complaint of finding a mass behind the left lower posterior teeth for 2 months. The mass did not cause obvious discomfort, and therefore, the patient did not seek medical treatment at the time. The patient felt that the mass had enlarged in the past two weeks, and there was slight pain on touching the site. The patient also experienced some episodes of pain on the left side of the neck. The patient reported no history of systemic diseases or drug allergy.

**Fig. 1:**
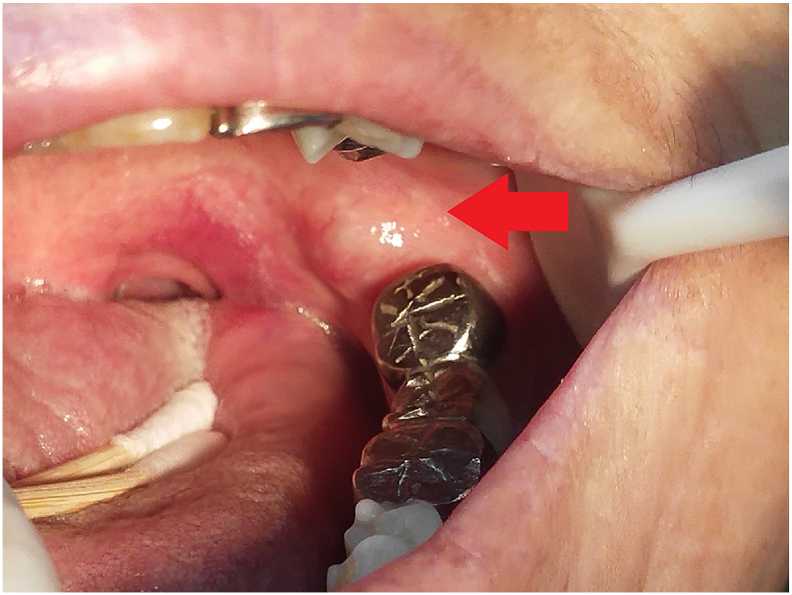
A 1 cm × 1 cm × 1 cm, round mass in the patient's left mandibular retromolar pad region is seen. The mucosal surface is smooth and intact, without congestion or erosion (red arrow). (For interpretation of the references to color in this figure legend, the reader is referred to the web version of this article.)

Clinical examination revealed the bilateral hemifacial contour was symmetric with normal skin color. The temporomandibular joints appear normal, and there is no tenderness around the muscles of mastication. Maximal mouth opening was 37 mm. Intraoral examination revealed a 1 × 1 cm, well-defined, round mass with tenderness in the left retromolar pad; slightly firm, with moderate mobility, the overlying mucosa was intact and normal in color without redness, swelling and erosion (Fig. 1). No obvious abnormality was found in other areas of the oral mucosa. The patient's tongue could move freely, and her mouth opening was unrestricted. Teeth 25–27 and 36–38 (FDI tooth numbering system) were restored by metal bridges. The submandibular and cervical lymph nodes were not palpable.

**Fig. 2:**
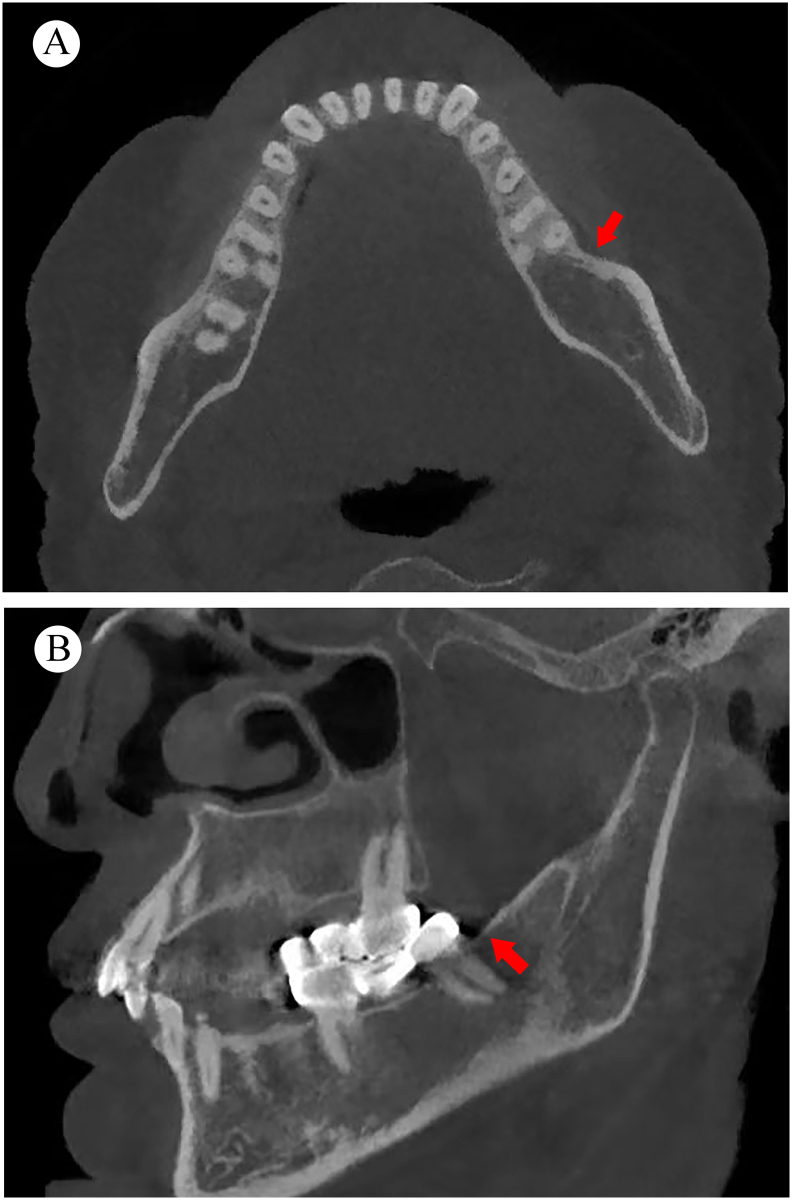
Cone beam computed tomography shows that the cortical bone of the left mandible is continuous without any obvious abnormality (red arrow). The axial plane (A) and the sagittal plane (B) images are shown here. (For interpretation of the references to color in this figure legend, the reader is referred to the web version of this article.)

Cone beam computed tomography (CBCT) shows that the cortical bone of the left mandible was continuous without any obvious abnormality (Fig. 2A–B). In addition, a low-density shadow around the distal root of tooth 38 was observed, indicating an inflammatory response in the periodontal areas; high density shadows are observed in the crown area of teeth 25–27 and 36–38 (Fig. 2B).

Based on its clinical manifestations, physical examination, and imaging examination, we got an initial impression of a benign mucocele. Since the mass was slightly firm with slight tenderness, and the patient felt it had enlarged, the possibility of a tumor, such as mucoepidermoid carcinoma, could not be ruled out. Further investigations were considered necessary to definitively diagnose the lesion. We resected the lesion with the patient under local anesthesia with the estimated surgical margin of 5 mm. The resected lesion specimen was then forwarded for pathological analysis.

**Fig. 3:**
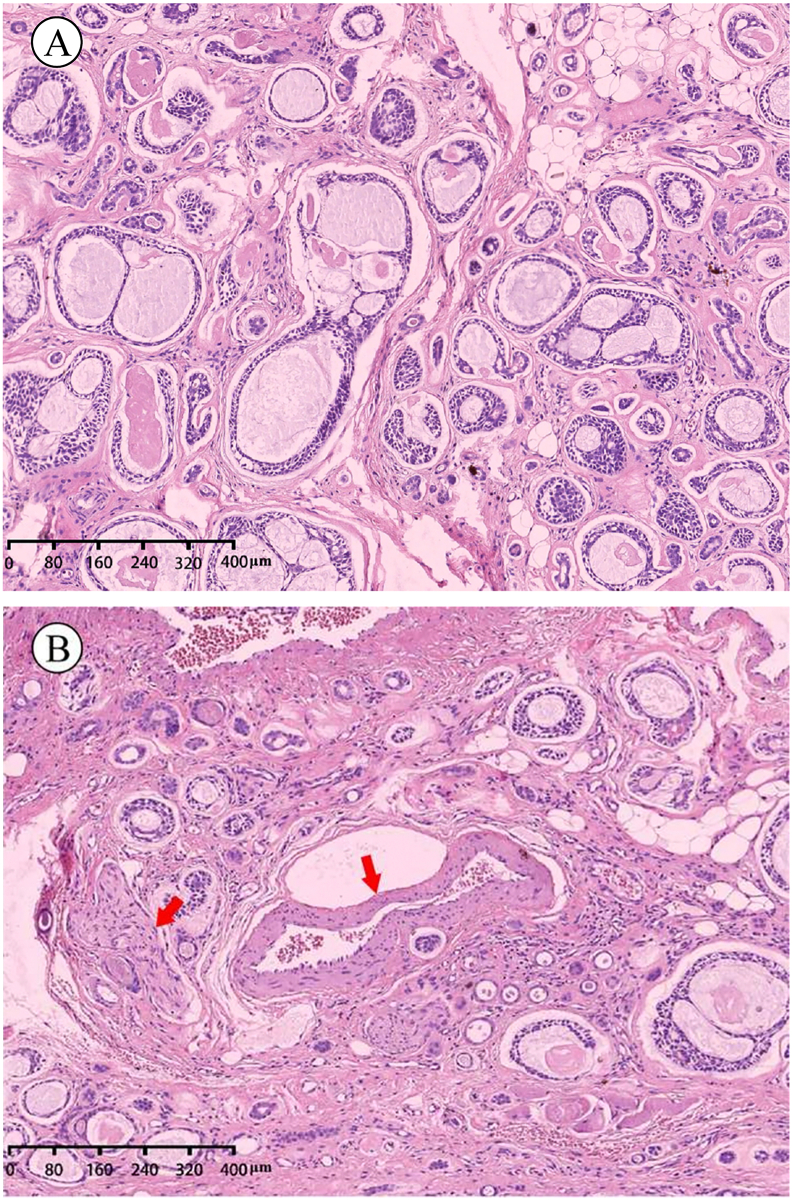
(A) Photomicrograph (Hematoxylin-Eosin staining) showing both cribriform and tubular tumor cell patterns. (B) Evidence of perineural invasion (red arrow, left) and vascular invasion is seen (red arrow, right). (For interpretation of the references to color in this figure legend, the reader is referred to the web version of this article.)

**Fig. 4:**
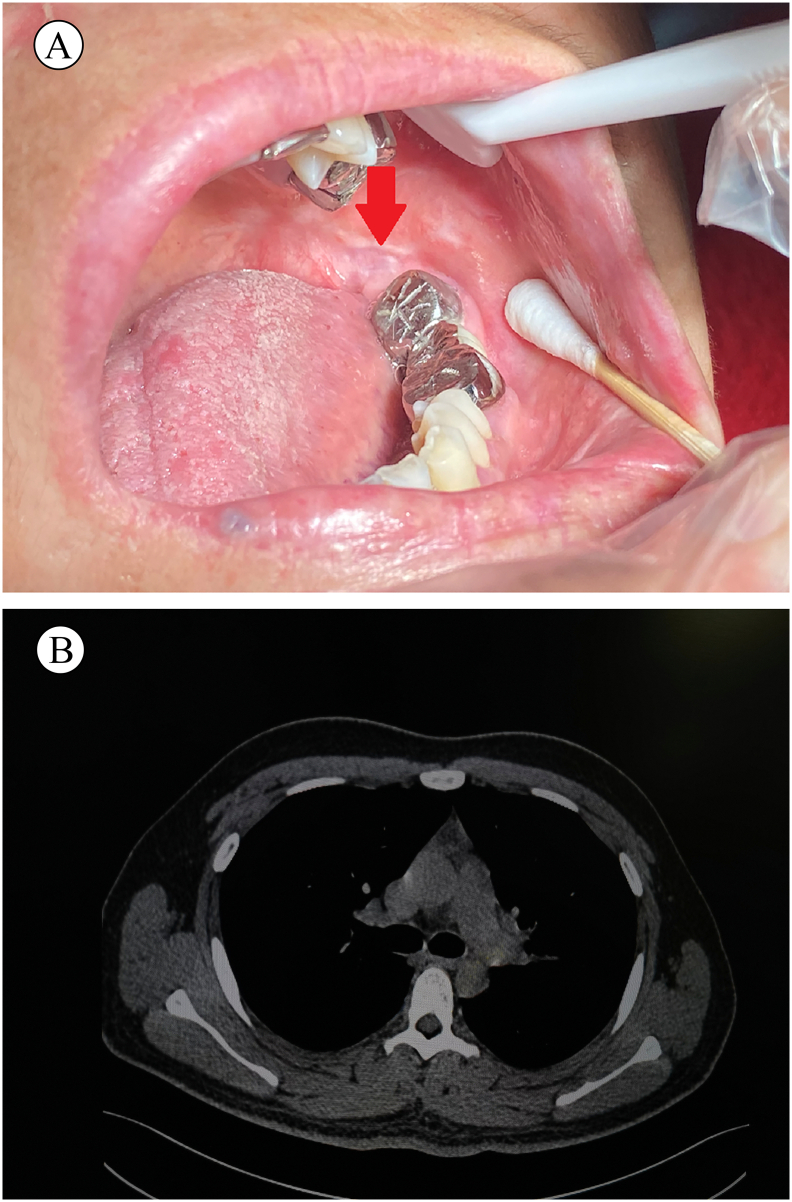
(A) Examination at the four-year follow-up reveals a smooth and intact mucosal surface (red arrow). A spiral CT scan of the lungs showed no abnormal metastatic lesions at two years (B). (For interpretation of the references to color in this figure legend, the reader is referred to the web version of this article.)

The pathological diagnosis was ACC (Fig. 3A). Grossly, the tumor was 1 cm × 1 cm × 0.6 cm, slightly firm, without a capsule, but it had a clear boundary. The sectioned surface of the tumor was solid and grey. Formalin-fixed, paraffin-embedded tissue sections were stained with hematoxylin and eosin for routine histologic examination. Microscopic observation revealed cystic cavities in the tumor parenchyma, which were cribriform or tubular, of different sizes, and filled with mucus. Cribriform and tubular cavities were lined with double layers of cells. The inner layer was formed by cubic or short columnar cells, and the outer layer was formed by fusiform cells. Tumor parenchymal cells invading the adjacent nerves and blood vessels were observed (Fig. 4B). No tumor cells were found at the surgical margin. A pathological diagnosis of ACC in the left mandibular retromolar pad region was established. Considering the histopathological findings, local extended resection of the lesion was recommended, but the patient did not agree. Therefore, close observation and regular follow-up were advised. The patient has been followed up for 4 years and 6 months. No local recurrence was detected, and the prognosis was good (Fig. 4).

## 3 Discussion

This paper provide a reference to avoid the clinical misdiagnosis of ACC in uncommon sites. When swelling occurs in the retromolar pad, particularly confined to the soft tissue, the list should include a ACC. Occasionally, the appearance of a ACC closely resembles a benign lesion, and it can be difficult to diagnose.

Mucoceles are mucous extravasation phenomena resulting from spontaneous ductal rupture or, less commonly, a traumatic cutting of a salivary excretory duct. Mucocele presents clinically as a painless swelling, bluish or translucent, and soft or freely movable [4,5]. Mucoepidermoid carcinoma is considered the most common malignant tumor affecting all of the salivary glands with varying proportions of mucinous, epidermoid, and intermediate cells, Most common presentation is painless swelling with pressure and discomfort, variably fixed, rubbery or soft [6,7]. Our case shows a round mass with tenderness; slightly firm, well-defined, with moderate mobility. Since mucoepidermoid carcinoma and mucocele are the most common clinically observed masses in the retromolar regions [8]. we initially considered the possibility of a mucocele based on the clinical and radiographic features but did not rule out the possibility of mucoepidermoid carcinoma. However, on pathological assessment, the lesion was confirmed to be ACC.

We conducted a literature review concerning the misdiagnosis of ACC and identified a few reports (Table 1). Dipen et al. [9] described a patient with ACC of the upper lip. Examination showed a painless, fluid-filled swelling with color ranging from bluish to black and soft in consistency, the patient was initially diagnosed as mucocele; Shogo and his colleagues reported a case of ACC with squamous differentiation. However, lesion was difficult to definitely diagnose as ACC owing to macroscopic and microscopic similarities to mucoepidermoid carcinoma. Finally, immunohistochemical analysis performed on HE slides confirmed the presence of ACC [10]. Similarly, ACC is easily misdiagnosed as other diseases. Zhao et al. [11] described a 43-year-old woman who presented with a persistent oral ulcer for approximately 1 year, the histopathological and immunohistochemical features were diagnostic of ACC. In addition, Ho provided a case report of parotid gland ACC in a 55-year-old female that was misdiagnosed as benign mixed tumor [12].

**Table 1:** Characteristics of 11 cases with a differential diagnosis between ACC and other diseases.

Author	Year	Age, yrs	Gender	Site	Initial diagnosis
Dipen et al. [9]	2023	36	M	Upper lip	Mucocele
Shogo et al. [10]	2015	54	F	Tracheobronchial	Mucoepidermoid carcinoma
Zhao et al. [11]	2013	43	F	Left upper molar gingiva	Ulcer
Ho et al. [12]	2023	55	F	Parotid gland	Benign mixed tumor
Saba et al. [13]	2014	56	M	Left floor of the mouth	Ulcer
Bárbara et al. [14]	2014	50	M	Maxillary sinus	Oroantral fistula
Medhini et al. [15]	2022	46	M	Soft palate	Palatal abscess
Vidya et al. [16]	2012	45	F	Left buccal mucosa	Benign soft tissue mass
Zhao et al. [17]	2023	57	M	Oropharynx	Chronic tonsillitis
Emre et al. [18]	2000	12	F	Tongue	Pleomorphic adenoma
Grimm et al. [19]	2012	55	F	Mandible	Apical periodontitis

ACC was first described by Billroth in 1859 as “cylindroma” due to the cylindrical arrangement of tumor cells in the hyaline stroma [20]. Cylindromas of the lacrimal and salivary glands are often associated with malignant behavior, such as invasiveness and metastasis, which is different from that of skin cylindromas. Therefore, cylindromas with malignant behavior are referred to as adenoid cystic carcinoma (ACC) to accurately describe their pathological features and clinical manifestations. Today, ACC is the most common term for the lesion [21]. As one of the most common epithelial malignant tumors of the salivary glands, ACC is highly invasive and neurophilic, easily infiltrates blood vessels, and rarely spreads to the lymph nodes [22]. ACC is most commonly found in the parotid and submandibular glands. It is also the most common cancer of minor salivary glands with the palate being the most common site. However, its occurrence in the minor salivary glands of the retromolar pad has not been reported in the literature [1,23,24]. Among the pathological subtypes of ACC, cribriform, tubular, and solid, the cribriform type is the most common, and the solid type is rare. But the latter has a higher rate of distant spread and lower rate of long-term survival [25,26].

Meanwhile, the correlation between nerve invasion and prognosis remains controversial. Some scholars believe that nerve invasion suggests increased risk of recurrence and distant metastasis [27], while Amit et al. [28] reported that nerve invasion is not correlated with distant metastasis. In this case, pathological examination exhibited both cribriform and tubular patterns, and solid-type ACC was not found. In addition, local perineural invasion and vascular invasion were seen under the microscope. The surgical margins had no tumor involvement. Therefore, local extended resection without cervical lymphadenectomy or postoperative radiotherapy was recommended. However, the patient was unwilling to undergo this treatment and was therefore instructed to closely observe the affected area and undergo regular follow-up.

## 4 Conclusion

Our review of the literature suggests that ACC of the minor salivary glands occurring in the retromolar pad is extremely rare, and it can be easily misdiagnosed based on clinical features alone. Therefore, clinicians should remain mindful of this rare and unusual condition during differential diagnosis and reach a definitive diagnosis based on a combination of clinical and pathological findings. Furthermore, long-term follow-up is particularly important for patients with ACC postoperatively.

## CRediT authorship contribution statement

All authors contributed equally.

## Declaration of competing interest

The author(s) declared no potential conflicts of interest with respect to the research, authorship, and/or publication of this article.
